# Myeloperoxidase to Risk Stratify Emergency Department Patients with Chest Pain

**Published:** 2009-06

**Authors:** Alex F. Manini, Andrew T. McAfee, Vicki E. Noble, J. Stephen Bohan

**Affiliations:** 1 *Harvard Affiliated Emergency Medicine Residency, Boston, MA, USA;*; 2 *Department of Emergency Medicine, Brigham and Women’s Hospital, Harvard Medical School, Boston, MA, USA;*; 3 *i3 Drug Safety, Auburndale, Massachusetts, USA;*; 4 *Department of Emergency Medicine, Massachusetts General Hospital, Harvard Medical School, Boston, MA, USA*

**Keywords:** chest pain, inflammation, myocardial infarction, peroxidase, risk

## Abstract

Previous studies suggest that serum myeloperoxidase (MPO) is a potentially useful biomarker to risk stratify troponin-negative patients with suspected myocardial ischemia. We hypothesized that the relationship between initial serum MPO levels would correlate with 30-day adverse cardiac outcomes for low risk emergency department (ED) patients with suspected myocardial ischemia. This prospective cohort study enrolled ED patients with chest pain or suspected myocardial ischemia, non-diagnostic ECG, and initially negative cardiac troponin I. We defined 30-day adverse cardiac events as death, myocardial infarction, or coronary revascularization. We calculated summary statistics, standard deviation (SD), odds ratios (OR), 95% confidence intervals (CI), and receiver operating characteristics (ROC). We enrolled 159 patients who had a mean age of 55 ± 13, were 56% female, of whom 5.2% suffered at least one adverse cardiac event. MPO test characteristics were poor, with an ROC area of only 0.47 (CI 0.23-0.71). MPO levels were not associated with adverse events (OR 0.99, CI 0.98-1.01, p=0.62). The optimal ROC cutpoint to predict adverse cardiac events had poor sensitivity and specificity (57% and 52%, respectively). Mean MPO concentrations in the event group did not differ from the non-event group. In this limited cohort of low risk ED patients with chest pain, we were unable to demonstrate utility of MPO for risk stratification. If confirmed in larger studies, these findings may call into question the routine use of MPO for low-risk chest pain.

## INTRODUCTION

Inflammatory events are implicated in all phases of the evolution of the coronary atherosclerotic plaque (1). Myeloperoxidase (MPO), a member of the heme-peroxidase superfamily, is a potential participant in the coronary atherogenic process (2). MPO-catalyzed reactions are linked with development of endothelial dysfunction (3), generation of atherogenic lipoproteins (4), and consumption of nitric oxide (5). Mechanistic links implicate MPO with the initiation and propagation of the mature atheroma (6), as well as complications of acute plaque rupture (7, 8).

In human subjects, MPO levels are correlated with angiographic evidence of atherosclerotic plaque (9). Serum MPO levels have prognostic value at 30-days and 6 months for emergency department (ED) patients with chest pain (10), as well as patients with acute coronary syndromes enrolled in the CAPTURE study (11).

The potential clinical utility of MPO for use in individual patients, while promising, remains unclear. Tests to assay serum MPO levels will soon become commercially available, but the test characteristics and timing of possible MPO elevation in acute coronary syndromes are unknown. Furthermore, low risk populations remain an important but as yet unstudied population of interest for potential risk stratification with MPO. A biomarker with high sensitivity would allow for potential safe discharge home for a large proportion of patients. We conducted a prospective, observational study to evaluate whether serum MPO levels predict 30-day adverse cardiac outcomes for low-risk ED patients with suspected myocardial ischemia.

## METHODS

### Selection and Description of Participants

This was a prospective, cohort study that analyzed the association between serum MPO levels and 30-day adverse cardiac events for low risk ED patients with suspected myocardial ischemia. The study and protocol for written informed consent were approved by the Institutional Review Board at the study institution.

The study institution was an urban, tertiary-care hospital with an annual census of approximately 55,000 adult visits. The ED was staffed by board-certified emergency physicians 24 hours per day.

Our population included a convenience sample (during business hours and only when research personnel were available) of low risk ED patients with chest pain or symptoms suggestive of myocardial ischemia. We defined low risk patients as those with non-diagnostic electrocardiogram (ECG) and negative initial cardiac markers (creatine kinase isoenzyme MB and cardiac troponin I). Patients with suspected unstable angina (based on the impression of the attending emergency physician), unstable vital signs, or any ECG changes consistent with acute ischemia or infarction were excluded.

Patients meeting inclusion criteria were approached for enrollment by either a research assistant or a co-investigator. Written informed consent was obtained for all subjects. Study staff members completed a questionnaire identifying demographics and cardiac risk factors.

Enrolled patients were admitted to the ED chest pain observation unit. All patients were pain-free on arrival to the unit and were placed on telemetry monitoring. Serial ECGs and cardiac markers (total creatine kinase, creatine kinase isoenzyme MB and troponin I) were performed over an 8 hour period. Patients with resolved symptoms and negative serial cardiac markers underwent either inpatient or outpatient stress testing at the discretion of the ED attending physician. Follow-up chart review of electronic medical records and a telephone call after 30 days were performed by one of the co-investigators.

Cardiac markers were measured using Bayer reagents (Bayer Healthcare, Cambridge, MA) on the Bayer Advia Centaur analyzer. Standard cutoff concentrations were used for total creatine kinase (41-266 units/liter), creatine kinase MB (0-5 ng/ml), and cardiac troponin I (0-0.10 ng/ml).

### Technical Information

Serum was drawn from patients upon completion of informed consent to the study. Serum acquisition occurred within 8 hours of arrival to the ED. No repeat samples were acquired. Blood was drawn into heparin-free tubes and centrifuged for ten minutes at 800 rpm. Plasma was separated from cells by pipetting into 500 micro-liter aliquots and stored at minus-20 degrees Celsius.

Plasma was shipped on dry ice to an independent, blinded laboratory (Oxford Biomedical Research, Oxford, MI) for measurement of MPO levels (in picomoles per liter, or pM) using the Oxis MPO-EIA kit (Oxis International, Inc., Foster City, CA). The MPO assay is a sandwich immunoassay to which plasma was added and used to measure the MPO concentration by detection of a fluorescence signal. Assay sensitivity was determined from eight individual measurements performed on standard curves from 4 separate runs, on the same day. The sensitivity signal was determined as 3.3 times the mean standard deviation of the 0 ng/ml standard from the individual curves. A linear extrapolation was made of this signal with the mean of 4 signal curves, and an interassay sensitivity was determined. Observed cross reactivity with eosinophil peroxidase is < 2%.

The composite outcome was 30-day adverse cardiac events, defined as any of the following: death, myocardial infarction (per the Joint European Society of Cardiology/American College of Cardiology criteria) (12), and coronary revascularization (defined as percutaneous coronary intervention or coronary artery bypass grafting).

### Statistics

To calculate anticipated sample size, we assumed a 5% incidence of adverse cardiac events (based on data from prior chest pain unit cohorts) (13-15), mean MPO levels of 80 (estimated standard deviation: 40) in the adverse event group and 50 (estimated standard deviation: 25) in the event free group (estimated from previous cohorts) (10-11). Using these assumptions with the “sampsi” command of STATA, we calculated the need to enroll 123 patients in the event-free group and 7 patients in the adverse event group to have 80% power (with 0.05 alpha) to detect a significant difference in mean MPO levels with the Student’s t-test.

For the primary analysis, Intercooled STATA (version 8.2) was utilized to calculate Fisher’s exact test (two-tailed) and Student’s t-test for categorical and continuous variables, respectively. Odds ratios (OR) based on univariate analysis, 95% confidence intervals (CI), and receiver operating characteristics (ROC) were also assessed (16). For our secondary data analysis, we defined the optimal MPO cutpoint as that which minimizes the difference between the false negative frequency and the false positive frequency (17). Using this cutpoint, we calculated the sensitivity and specificity of MPO for 30-day adverse cardiac events. All p values were two-tailed with one degree of freedom, with a value <0.05 considered significant.

## RESULTS

### Clinical Characteristics

Of 159 patients meeting inclusion criteria, 24 patients (15%) were lost to follow-up, yielding 135 patients (85%) for data analysis. The clinical characteristics for all patients evaluated in the study are shown in Table 1. Patients lost to follow-up tended to be younger, but otherwise had similar clinical characteristics to those with follow-up data (Table 2).

**Table 1:** Baseline clinical characteristics

*Characteristic*	*All Patients No.(%) (n=135)*	*Patients with adverse events No.(%) (n=7)*	*Patients without adverse events No.(%) (n=128)*	*p Value*

Mean Age (+/**-** SD)	54.9 +/**-** 12.8	52.9 +/**-** 9.9	55 +/**-** 12.98	NA
Male Gender	60 (44)	4 (57)	56 (44)	NA
Cocaine Use Within 24 Hours	2 (1)	0 (0)	2 (2)	NA
Prior MI	7 (5)	1 (14)	6 (5)	NA
*Cardiac Risk Factors*				
Current Smoker	23 (17)	2 (29)	21 (16)	NA
Hypertension	62 (46)	4 (57)	58 (45)	NA
Hypercholesterolemia	53 (39)	2 (29)	51 (40)	NA
Family History	45 (33)	4 (57)	41 (32)	NA
Diabetes Mellitus	21 (16)	2 (29)	19 (15)	NA
*Cardiac Markers*				
Mean Initial Total CK (U/L)	119.5	148.6	117.9	0.31†
Mean Serial^b^ Total CK (U/L)	121.6	464.0	102.1	**<0.0001**^a^
Mean Initial MB (ng/ml)	1.6	2.5	1.5	**<0.05**^a^
Mean Serial^b^ MB (ng/ml)	3.3	39.6	1.25	**<0.0001**^a^
Mean Initial Troponin I (ng/ml)	<0.01	<0.01	<0.01	1.0^a^
Mean MPO (pM)	78.3	61.7	79.2	0.62^a^
Mean ACI-TIPI Score	17.6	19.7	17.5	0.73^a^
TOTAL	135 (100)	7 (5.2)	128 (94.8)	

Significant p values are in bold;

a Student t-test for continuous variables;

b Serial serum measurements indicate results of the second set drawn after 8 hours. SD, standard deviation; MI, myocardial infarction; CK, creatine kinase; MB, creatine kinase isoenzyme MB; MPO, myeloperoxidase; ACI-TIPI, acute cardiac ischemia time insensitive predictive instrument; ng, nanograms; mg, milligrams; U, units; L, liter; ml, milliliter; pM, picomolar; NA, not applicable.

**Table 2:** Characteristics of patients lost to follow-up

*Characteristic*	*All Patients No.(% or SD)*	*Patients LTFU No.(% or SD)*	*Patients With Follow-up No.(% or SD)*	*p Value*

Mean Age (+/**-** SD)	53.9 (12.5)	48.0 (8.3)	54.9 (12.8)	**0.011**^c^
Male Gender	75 (47)	15 (63)	60 (44)	0.12^a^
Cocaine Use Within 24 Hours	3 (2)	1 (4)	2 (2)	0.39^a^
Prior MI	7 (4)	0 (0)	7 (5)	0.60^a^
*Cardiac Risk Factors*				
Current Smoker	26 (16)	3 (13)	23 (17)	0.77^a^
Hypertension	74 (47)	12 (50)	62 (46)	0.71^b^
Hypercholesterolemia	64 (40)	11 (46)	53 (39)	0.55^b^
Family History	54 (34)	9 (38)	45 (33)	0.82^a^
Diabetes Mellitus	24 (15)	3 (13)	21 (16)	1.00^a^
*Cardiac Markers*				
Mean Serial^d^ Total CK (U/L)	125.2 (206.4)	144.7 (87.4)	121.6 (221.6)	0.62^c^
Mean Serial^d^ MB (ng/ml)	3.01 (19.9)	1.46 (0.89)	3.29 (21.7)	0.68^c^
Mean Initial Troponin I (ng/ml)	<0.01	<0.01	<0.01	1.0^c^
Mean MPO (pM)	83.4 (106.4)	111.9 (173.2)	78.3 (89.6)	0.15^c^
Mean ACI-TIPI Score	16.8 (15.3)	12.7 (13.5)	17.6 (15.5)	0.17^c^
TOTAL	159 (100)	24 (15)	135 (85)	

Significant p values are in bold;

a Fisher’s exact test or

b chi squared test for categorical variables.

c Student t-test for continuous variables;

d Serial serum measurements indicate results of the second set drawn after 8 hours. LTFU, lost to follow-up; SD, standard deviation; MI, myocardial infarction; CK, creatine kinase; MB, creatine kinase isoenzyme MB; MPO, myeloperoxidase; ACI-TIPI, acute cardiac ischemia time insensitive predictive instrument; ng, nanograms; mg, milligrams; U, units; L, liter; ml, milliliter; pM, picomolar.

Cardiac markers associated with adverse events included mean initial creatine kinase isoenzyme MB levels (p=0.049), mean serial creatine kinase levels (p<0.0001), and mean serial creatine kinase isoenzyme MB levels (p<0.0001). There was not a statistically significant difference between mean MPO levels for patients with (61.7 pM, SD 47.5, CI 37.6-86.1) and without (79.2 pM, SD 91.4, CI 63.4-96.8) adverse events (p=0.62).

### Power Analysis

Due to the unexpectedly limited differences in mean MPO levels between groups with and without adverse events, the present study likely did not achieve the 80% power which was calculated *a priori*. We therefore performed a sensitivity analysis on the patients lost to follow-up. Assuming the worst case scenario in which everyone lost to follow-up had an adverse event (i.e. death, MI, or revascularization), the MPO levels were not significantly different between groups (94.0 ± 23.5, CI 46.3-141.6, t-test p=0.3).

### Distribution of MPO Levels

**Figure 1:**
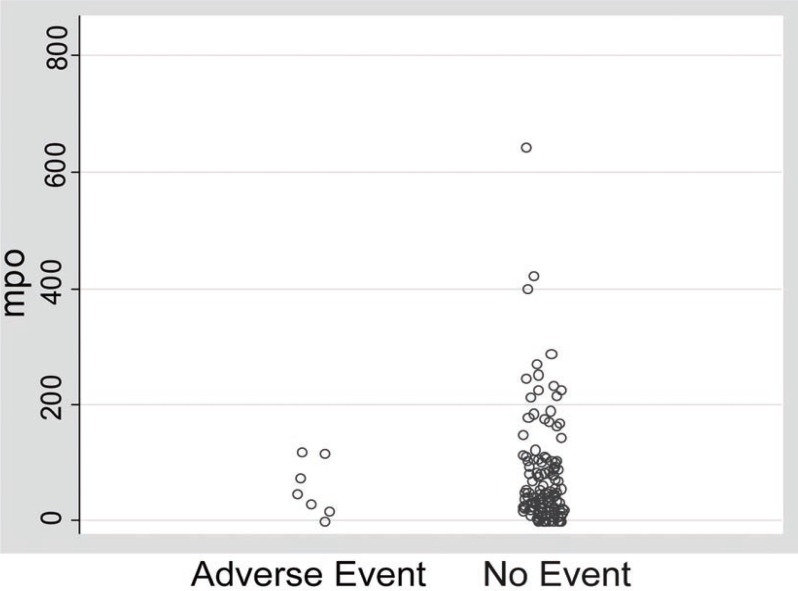
MPO distribution comparing patients with and without adverse cardiac events. This jitterplot visually demonstrates the MPO distribution of all patients in the study. The MPO level for each individual patient is represented by a single oval. Patients with a 30-day adverse cardiac event are grouped to the left, compared with all other patients on the right. There was not a useful MPO cutpoint to arbitrarily separate patients with events from those without events. MPO, myeloperoxidase (picomolar).

The distribution of MPO results is visually demonstrated in Figure 1. Based on the finding that MPO levels were relatively low in patients with adverse events, there was not a useful cutpoint to arbitrarily separate patients with events from those without events. There were only 13 patients (5.4% of entire cohort) in the event-free group whose MPO levels were lower than all patients in the adverse event group.

### Clinical Follow-up

Patients with adverse events are summarized in Table 3. The 30-day incidence of adverse cardiac events in the study population was 5.2%. There were no deaths in the study population within 30 days. There were 7 patients with at least one adverse cardiac event (among these 7 patients, there were 4 myocardial infarctions, 4 percutaneous coronary interventions, and 1 coronary artery bypass graft).

**Table 3:** Summary of adverse cardiac events^a^

Patient	MPO (pM)	ED Course	Inpatient	30-Day Follow-up

1	11.7	NSTEMI	LAD Stent	Alive
2	19.9	Positive STRESS	LAD Stent	Alive
3	22.0	STEMI	LAD Stent	Alive
4	50.1	UAP	LAD Stent	Alive
5	89.3	SVT	NSTEMI	Alive
6	111.2	NSTEMI	LAD Stent	Alive
7	127.6	Positive STRESS	CABG	Alive

a The seven patients with 30-day adverse cardiac events are shown in order of ascending MPO level. For each patient, any adverse events during the ED course, inpatient course, and 30-day follow-up are listed. MPO, myeloperoxidase; pM, picomolar; ED, emergency department; NSTEMI, non-ST elevation myocardial infarction; STEMI, ST-elevation myocardial infarction; UAP, unstable angina pectoris; SVT, supraventricular tachycardia; CABG, coronary artery bypass graft; LAD, left anterior descending coronary artery; LCX, left circumflex coronary artery.

### Univariate Analysis

Results of a univariate analysis for the association between cardiac markers and adverse cardiac events are summarized in Table 4. Of the cardiac markers evaluated, only serial creatine kinase isoenzyme MB levels (OR 1.7, CI 1.08-2.65, p<0.03) significantly predicted 30-day adverse events, while initial creatine kinase isoenzyme MB levels showed a trend toward significance (OR 1.5, CI 0.97-2.2, p<0.08). MPO levels did not predict adverse events (OR 0.99, CI 0.98-1.01, p = 0.62). No demographic factors (age, sex) or cardiac risk factors (hypertension, diabetes, hypercholesterolemia, family history, or current smoking) were statistically significant predictors of 30-day adverse cardiac events (data from univariate analysis not shown).

**Table 4:** Univariate analysis of cardiac markers as predictors of adverse events^a^

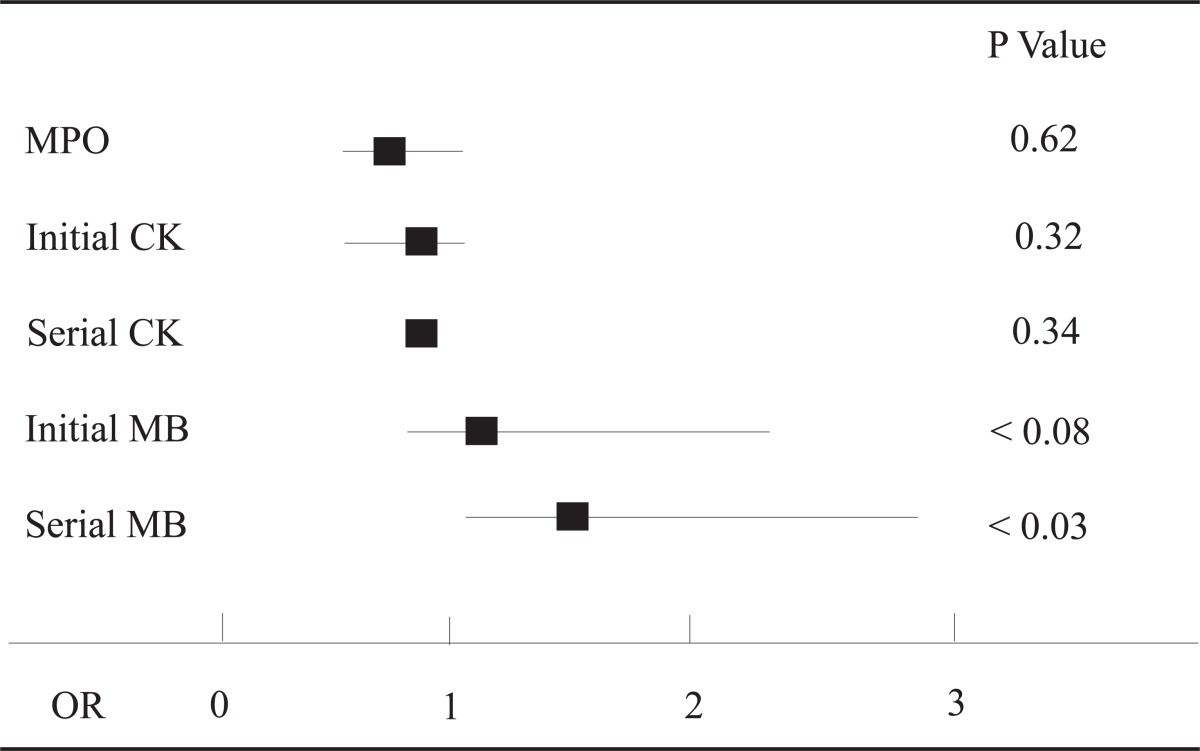

a Odds ratios (OR) are shown for each cardiac marker as a predictor of 30-day adverse cardiac events. The OR is represented as a filled box, and the 95% confidence interval is represented as a line. MPO, myeloperoxidase; CK, creatine kinase; MB, creatine kinase MB isoenzymes; OR, odds ratio.

### Receiver Operating Characteristics

**Figure 2:**
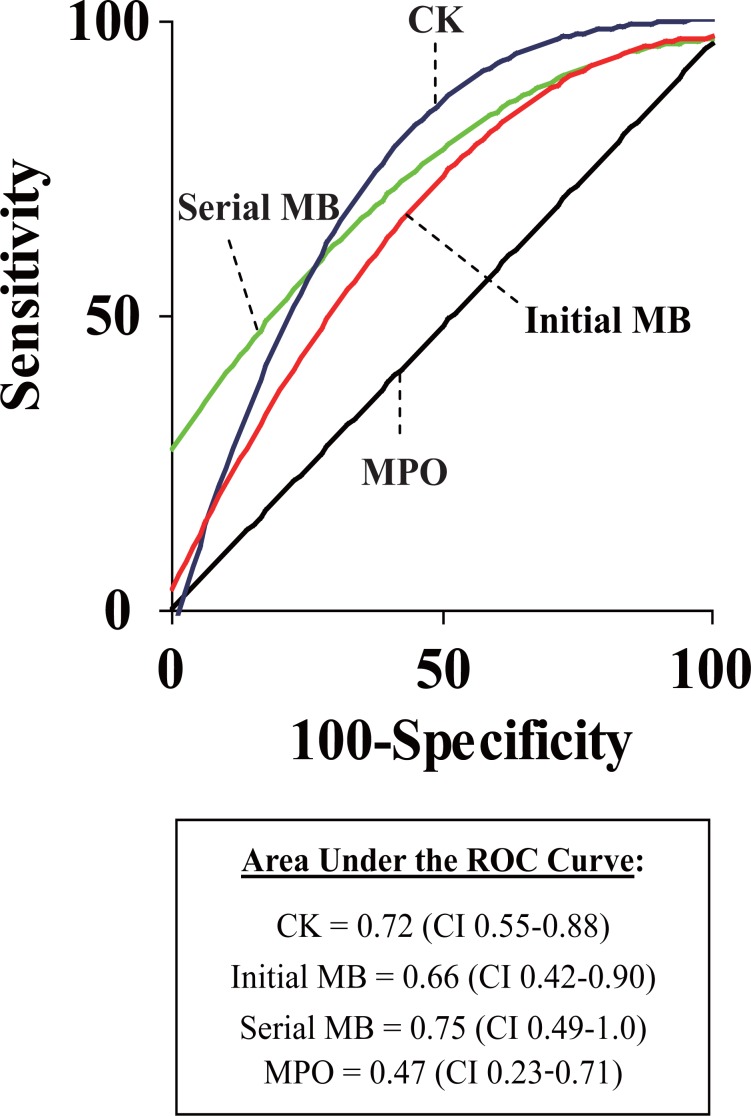
Receiver operating characteristics for cardiac markers to predict 30-day adverse events. Shown are ROC curves for the initial creatine kinase (CK), initial creatine kinase MB isoenzyme (Initial MB), serial creatine kinase MB isoenzyme (Serial MB), and base-line values of myeloperoxidase (MPO). The true positive fraction (sensitivity) is plotted against the false positive fraction (1 - sensitivity) to quantitate the diagnostic accuracy of each biologic marker. Areas under the ROC curves were as follows: CK = 0.72 (CI 0.55-0.88), Initial MB = 0.66 (CI 0.42-0.90), Serial MB = 0.75 (CI 0.49-1.0), MPO = 0.47 (CI 0.23-0.71). ROC, receiver operating characteristics; CK, creatine kinase; MB, creatine kinase isoenzyme MB; MPO, myeloperoxidase; CI, 95% confidence intervals.

The receiver operating characteristics (ROC) of various cardiac markers as predictors of 30-day adverse cardiac events are demonstrated in Figure 2. Test characteristics for MPO as a predictor of adverse events were poor, with an ROC curve area of only 0.47 (CI 0.23-0.71). In contrast, serial creatine kinase isoenzyme MB was more predictive than MPO, with an ROC curve area of 0.75 (CI 0.49-1.0).

### Test Characteristics of MPO Cutpoint

In order to calculate the sensitivity and specificity of MPO, we designated an optimal cutpoint. We chose the cutpoint (46.7 pM) as the MPO level which minimized the difference between the false positive frequency and the false negative frequency for 30-day adverse cardiac events (17). At this cutpoint, the sensitivity and specificity were 57% and 52%, respectively, with a likelihood ratio positive (LR+) of 1.18 and a likelihood ratio negative (LR-) of 0.83.

## DISCUSSION

In our low-risk population, MPO was not associated with 30-day adverse cardiac events. There was no difference in mean MPO levels between patients with and without adverse cardiac events in our study (Table 1). Due to the relatively low distribution of MPO levels in the adverse events group (Figure 1), there was no threshold under which a significant subset of patients at low or no risk for events could be identified. Using univariate analysis (Table 4), MPO did not predict adverse outcomes (OR 0.99, CI 0.98-1.01, p=0.62).

### Prior Studies

Previous studies suggested that MPO may have utility for risk stratification of ED patients with chest pain and patients with acute coronary syndromes. Brennan and colleagues (10) evaluated 604 consecutive patients presenting with chest pain to the emergency department and found that for all patients, increasing quartiles of MPO carried increased risk (4^th^ quartile OR 4.7) of major adverse cardiac events (myocardial infarction, re-infarction, revascularization, and death) at 30 days and 6 months. Similarly, Baldus and colleagues (11) evaluated 1090 patients with acute coronary syndromes enrolled in the CAPTURE (18) trial and found that MPO serum levels were predictive of subsequent death and myocardial infarction at 6 months (adjusted hazards ratio 2.25 between patients with high vs. low MPO). Remarkably, for individuals in whom cardiac troponin T was initially negative, an elevated MPO level at the time of presentation was predictive of subsequent major adverse cardiac events in both studies. Neither of the above studies reported sensitivity or specificity of an MPO cutpoint.

### MPO Risk Stratification

In our study of low risk ED patients with suspected myocardial ischemia, the test characteristics for MPO were poor (Figure 2), and MPO performed worse than two markers of myocardial necrosis (i.e., creatine kinase and creatine kinase isoenzyme MB). Furthermore, selection of an MPO cutpoint using standard techniques for clinical decision making (17) yielded disappointing sensitivity and specificity. Despite likely being under-powered (due to a smaller than expected difference in mean MPO levels between groups), a sensitivity analysis assuming a “worst case” scenario did not change the results of the data analysis. These provocative results suggest that MPO has poor utility for individual ED patients with chest pain.

We studied MPO concentrations within 8 hours of ED presentation, but it is currently unknown whether the timing of MPO rise has any clinical significance, as it is not a marker ischemia but rather of inflammation. Previous studies (10, 11, 18) assessing serum MPO in patients with chest pain analyzed concentrations within 18-24 hours of presentation or at the time of cardiac catheterization. One study in low-risk subjects (19) assessed MPO as part of a multi-marker panel in the chest pain unit, but exact timing of blood measurements were not specified. Therefore, our study represents the earliest time at which MPO levels have been assessed for patients with acute chest pain.

One can argue the value of testing a high risk cohort (as has been done previously), however testing a low risk cohort, as we have done in this study, may have more clinical relevance. Mitchell and colleagues (19) used a multi-marker approach in low risk subjects and also found that MPO had poor predictive utility for acute coronary syndromes within 45 days. Our goal was to obtain objective evidence of plaque instability or ischemia in order to justify admission. We found no correlation for MPO, which is of interest because it conflicts with work from prior studies.

Our data do not support the use of MPO for risk stratification of a low risk ED patient population with suspected myocardial ischemia. By use of the Student’s t-test, ROC analysis, univariate analysis, and examination of an optimal cutpoint, we found little support for MPO as a useful biomarker to predict short term cardiac outcomes for these patients.

### Limitations

The analysis of this data set has several important limitations. Selection of non-consecutive patients from only one institution, and the low incidence of adverse cardiac events in the study cohort may limit conclusions about external validity. While our primary analysis was likely underpowered, our sensitivity analysis did not change the conclusions of this study. Our composite endpoint did not include unstable angina, which may increase the association between MPO levels and cardiac outcomes. Furthermore, use of an independent lab to perform the MPO assay limits the generalizability of these results.

Finally, the timing of any possible MPO elevation during acute coronary syndromes is unknown. We drew serum within the first 8 hours of presentation to the ED, but time of onset of chest pain is not recorded and more timely or serial blood draws during the ED course may have enhanced detection of an association between MPO and adverse events. To avoid these limitations, future studies might benefit from a larger, consecutive sample taken from multiple hospital centers with comparison between initial and serial serum MPO levels.

### Conclusions

In this small cohort of ED patients with chest pain but non-diagnostic ECG and initially negative cardiac troponin I, serum MPO levels were not associated with 30-day adverse cardiac events. Larger, multi-center studies are warranted to confirm these findings.
